# Identification and characterization of *ORF19.1725*, a novel gene contributing to the white cell pheromone response and virulence-associated functions in *Candida albicans*

**DOI:** 10.1080/21505594.2018.1456228

**Published:** 2018-05-04

**Authors:** Fu-Sheng Deng, Ching-Hsuan Lin

**Affiliations:** Department of Biochemical Science and Technology, College of Life Science, National Taiwan University, Taipei, Taiwan

**Keywords:** Candida albicans, opaque cells, ORF19.1725, pheromone response, virulence, white cells

## Abstract

An epigenetic transition between white cells and opaque cells influences several properties of *Candida albicans* biology, including cellular morphology, biofilm formation, virulence, and sexual mating. In particular, these two cell types exhibit marked differences in their ability to undergo sex. A previous study identified the transcriptional regulator of pheromone response in both the white and opaque states as Cph1 because deletion of this gene abolished both pheromone-induced cell adhesion in white cells and sexual mating in opaque cells. To further explore how these cell types exhibit distinct biological outputs upon pheromone stimulation, we selected five Cph1-regulated genes with significant expression during the pheromone response in the white state but not the opaque state. These phase-specific pheromone-induced genes are *ORF19.1539, ORF19.1725, ORF19.2430, ORF19.2691* and *ORF19.5557*. Deletion of each gene revealed that *orf19.1539Δ, orf19.1725Δ, orf19.2430Δ* and *orf19.5557Δ* showed significant decreases in pheromone-stimulated cell adhesion in the white state but retained normal mating competency in the opaque state, indicating that a particular role in white cell pheromone response is mediated by these four genes. Interestingly, the defects of *orf19.1725Δ* in pheromone-stimulated cell adhesion also abolished conventional biofilms and hyphal growth. Zebrafish egg infection assays further demonstrated that *ORF19.1725* is involved in cell adhesion, penetration and virulence. Overall, four Cph1-regulated downstream targets were identified in the regulation of white cell pheromone response. We also clarified the roles of *C. albicans ORF19.1725* in cell adhesion, hyphal growth, biofilm formation and virulence.

## Introduction

*Candida albicans* can be a harmless, normal member of the microbiota in and on the human body [1]. However, it is also an opportunistic pathogen that can attack multiple locations in a human host, including the skin, genitals, mucous membranes and kidneys, possibly resulting in life-threatening systemic infection or invasive candidiasis [2,3]. The propensity of *C. albicans* to adapt and become pathogenic in different niches has been closely linked to its phenotypic plasticity [4,5]. Indeed, several diverse *C. albicans* cell types, including yeast cells, hyphal cells, pseudohyphae, chlamydospores, GUT (gastrointestinally induced transition) cells, gray cells and opaque cells [6-9], demonstrate the ability of *Candida* cells to alter their morphology and behavior in response to environmental signals.

Among these cellular alterations, the reversible morphological transition between white cells and opaque cells is particularly interesting, because these two morphologically distinct cell types exhibit a wide range of different biological behaviors. Typically, white cells are mating incompetent, can release immune-cell chemoattractants and exhibit greater virulence in systemic infections, whereas opaque cells can be fertilized and cause more serious infections of mammalian skin [10-12]. Furthermore, white and opaque cells produce different sets of chemoattractants, thereby affecting *C. albicans* interactions with phagocytic cells [13–18]. Specifically, white cells release Sap2, Sap6 and an undetermined low-molecular-mass peptide as chemoattractants and are recognized by phagocytes, whereas opaque cells produce Sap1, Sap2 and Sap3 and are able to escape phagocytosis [13,14,18]. Additionally, transcriptional profiling has shown that white cells and opaque cells display large-scale differences in the expression of metabolic genes [19,20]. Indeed, a very recent report has shown that the white cell state is an “intrinsic phenotype”, allowing this cell type to grow better at high temperatures (37°C) in mammalian hosts and in response to many nutritional conditions and chemical stresses [21]. However, opaque cells show greater fitness than while cells under poor nutrition conditions and in certain environmental circumstances [21]. Despite the higher fitness of white cells under a wide range of environmental conditions, several environmental stimuli, including oxidative stress, CO_2_ and N-acetylglucosamine (NAG), induce opaque cell formation [22–24].

The regulation of epigenetic switching between the white and opaque states in *C. albicans* involves a complicated interlocking transcriptional feedback loop of eight factors, Wor1, Wor2, Wor3, Wor4, Czf1, Efg1, Ahr1 and Ssn6 [19,20,25–29]. This network is also controlled by the **a**1/α2 heterodimer protein generated by **a**/α cells, which leads to the repression of white-to-opaque switching [30].

One of the most interesting differences between white cells and opaque cells is their behavior during pheromone response, in which, unlike opaque cells, white **a** or α cells do not undergo a mating response but display increased cell adhesion (pheromone-stimulated cell adhesion) and sexual biofilm development [31,32]. In addition, it has been suggested that sexual biofilm formation by white cells provides an optimal condition for opaque cell mating [33]. Genetic analyses have defined the mechanism by which pheromone signaling activates cellular responses in white and opaque cells. In particular, both cell types, when challenged with pheromone, are regulated by the same signaling pathway and the same transcription factor, Cph1 [31]. A key question, then, is how *C. albicans* displays distinct functions upon exposure to the same pheromone signal while still utilizing the same transcription factor. It is possible that some downstream genes regulated by Cph1 may be different in white and opaque cells after pheromone treatment, leading to different responses in these two cell types. Interestingly, Cph1 is not required for the formation of “conventional biofilms” [31]. Conventional biofilms, which are chiefly regulated by six transcription factors in *C. albicans* (Tec1, Efg1, Rob1, Ndt80, Bcr1 and Brg1) [34], are formed when *C. albicans* yeast cells adhere to a surface, form pseudohyphae and hyphae and produce extracellular matrix materials [35,36]. Thus, the genetic control of these two distinct biofilm types in *C. albicans* is mediated by different mechanisms [31,32].

In this study, we used previous transcriptional profiling data [31] to identify five novel downstream genes that are highly regulated by Cph1 in the *C. albicans* white state when challenged with pheromone but are less regulated or unaffected by Cph1 and pheromone challenge in the opaque state. We therefore hypothesized that these genes might play a specific role in white cell pheromone response. Deletion of each gene revealed that most of the mutant strains showed effects on pheromone-stimulated cell adhesion in white cells. In particular, *orf19.1539Δ, orf19.1725Δ, orf19.2430Δ* and *orf19.5557Δ* showed significant reductions in cell adhesion during pheromone response. Interestingly, *ORF19.1725* is also involved in virulence-associated functions, including the formation of conventional biofilms, hyphal formation, cell adhesion and virulence. Overall, our data reveal novel genes that specifically contribute to white cell pheromone response. These results provide a clue to the mechanism that allows white and opaque cells to display distinct pheromone responses and behaviors.

## Results

### Screening for white-specific and Cph1-regulated genes in pheromone-challenged P37005

**Figure 1.**
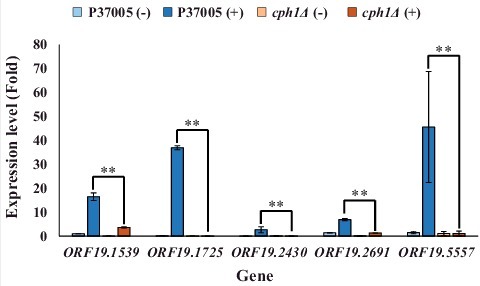
Quantitative RT-PCR demonstrated that expression of the *ORF19.1539, ORF19.1725, ORF19.2430, ORF19.2691* and *ORF19.5557* genes in white cell pheromone response were regulated by Cph1 in P37005. Expression was analyzed and compared between the wild-type and *cph1Δ* with or without α-pheromone peptide treatment. Expression was normalized to that of the *ACT1* gene. Values are the mean ± SD of three experimental replicates, and two technical repeats were performed for each experimental replicate. **: *P* < 0.01.

Transcriptional profiling has shown that many pheromone response genes are identical in both white and opaque cells [31]. To further explore the regulatory differences between the white and opaque states during pheromone response, we screened genes that were significantly upregulated by Cph1 in white cells. Five highly expressed genes in the white state showed significant differences (*P* < 0.05) in expression fold change between white cells and opaque cells of the wild-type strain during the response to α-pheromone [31]. These genes were *ORF19.1539, ORF19.1725, ORF19.2430, ORF19.2691* and *ORF19.5557* (*MNN4*). To confirm these results, quantitative PCR was performed. As shown in Fig. 1, all candidate genes were highly expressed during pheromone response in the white state. However, the expression level of each gene was significantly reduced in the P37005 *cph1Δ* strain after pheromone treatment. These results showed that Cph1 is a key regulator and is required for the expression of these five genes in white cell pheromone response. Among them, only *MNN4* has a known function, and it has not been studied in depth; this gene encodes a mannosyltransferase and is involved in the cell wall formation and engulfment process during phagocytosis in *C. albicans* [37–39]. The functions of the other selected genes are entirely unknown, and none have been studied previously.

### *orf19.1539Δ, orf19.1725Δ, orf19.2430Δ* and *orf19.5557Δ* reduced cell adhesion ability in white cells during pheromone response but were dispensable in cellular mating of opaque cells in P37005

**Figure 2.**
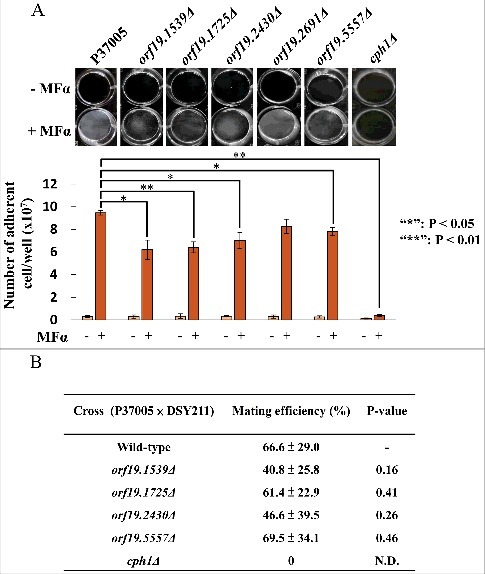
*ORF19.1539, ORF19.1725, ORF19.2430* or *ORF19.5557* in *C. albicans* P37005 are specifically involved in white cell pheromone response but are dispensable in opaque cell pheromone response. (A) White cells of P37005 cultured in Lee's medium in the presence or absence of pheromone (MFα) in plastic dishes. Images were taken after PBS washing (top panel), and the remaining adhered cells were quantitated (bottom panel). (B) Opaque cells of the wild-type and mutant strains (*MTL*a/a; SAT^−^, Arg^+^) were crossed with the *MTL*α/α DSY211 strain (SAT^+^, Arg^−^) on Spider medium for 48 hr. Cells were plated onto selective media (SAT^+^, Arg^−^) to quantitate mating efficiencies. The *cph1Δ* strain of P37005 served as a negative control. Values are the mean ± SD of three experimental replicates, and two technical repeats were performed for each experimental replicate. *: *P* < 0.05; **: *P* < 0.01.

To understand their functions in pheromone response, each gene was deleted by using a target recombination strategy, as described in the Materials and Methods. Then, the pheromone response of white cells was investigated to see whether the deletion strains were defective in cell adhesion. Fig. 2A shows that compared to the P37005 wild-type strain, the adherent numbers of *orf19.1539Δ, orf19.1725Δ, orf19.2430Δ* and *orf19.5557Δ* were 6.2 × 10^7^, 6.4 × 10^7^, 7.0 × 10^7^ and 7.8 × 10^7^, respectively, significantly lower than that of wild-type strain P37005 (9.47 × 10^7^). Pheromone responses between the white and opaque states are typically distinct. Opaque cells mate; white cells, however, generate a sexual biofilm and promote cell adhesion [31–33]. To further determine whether these mutant strains defective in white cell pheromone response also showed phenotypes in opaque cell pheromone response, mating assays were performed. Interestingly, the *orf19.1539Δ, orf19.1725Δ, orf19.2430Δ* and *orf19.5557Δ* strains displayed normal **a**-α mating efficiencies (Fig. 2B). These results indicated that these four genes are specifically upregulated in *C. albicans* white cells in order to execute precise biological responses.

### *ORF19.1725* is required for conventional biofilm development

**Figure 3.**
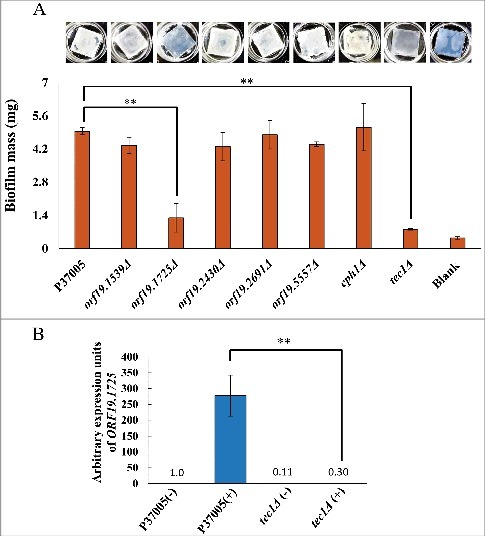
*ORF19.1725* is also regulated by Tec1 and is required for the formation of conventional biofilms. (A) Analysis of five candidate genes in a conventional biofilm assay on silicone squares revealed that the presence of the *ORF19.1725* gene is necessary for the development of conventional biofilms. The graph shows the mean ± SD of three experimental replicates, and two technical repeats were performed for each experimental replicate. “**”: *P* < 0.01. (B) Quantitative RT-PCR revealed that *ORF19.1725* expression was regulated by Tec1. Expression was analyzed and compared between the wild-type and *tec1Δ* in response to pheromone treatment. Expression was normalized to that of the *ACT1* gene. Values are the mean ± SD of three experimental replicates, and each replicate represents two technical repeats. “**” represents *P* < 0.01. – and + indicate with or without pheromone treatment, respectively.

Previous works have demonstrated that Cph1 is not involved in conventional biofilms [31]. Instead, the conventional biofilm regulatory network is modulated by Tec1 and five other major transcription factors [34]. Loss of any one of these regulators significantly compromises biofilm formation *in vitro* and *in vivo* [34]. To further investigate the five candidate white-specific pheromone response genes in P37005, we also tested their roles in conventional biofilm development. Among them, one gene significantly influenced biofilm formation on silicone, *ORF19.1725*, as deletion of this gene led to a biofilm mass of 1.3 mg, whereas the biofilm mass of wild-type strain P37005 was 4.95 mg (Fig. 3A). This result agrees with previously published microarray data, in which *ORF19.1725* was regulated by both Cph1 and Tec1 [31,40]. Indeed, quantitative RT-PCR confirmed the regulation of *ORF19.1725* by Tec1 (Fig. 3B), a transcription factor required for the formation of conventional biofilms but not for pheromone-stimulated cell adhesion [31,34].

### *ORF19.1725* deletion caused a severe defect in hyphal formation in *C. albicans* P37005

**Figure 4.**
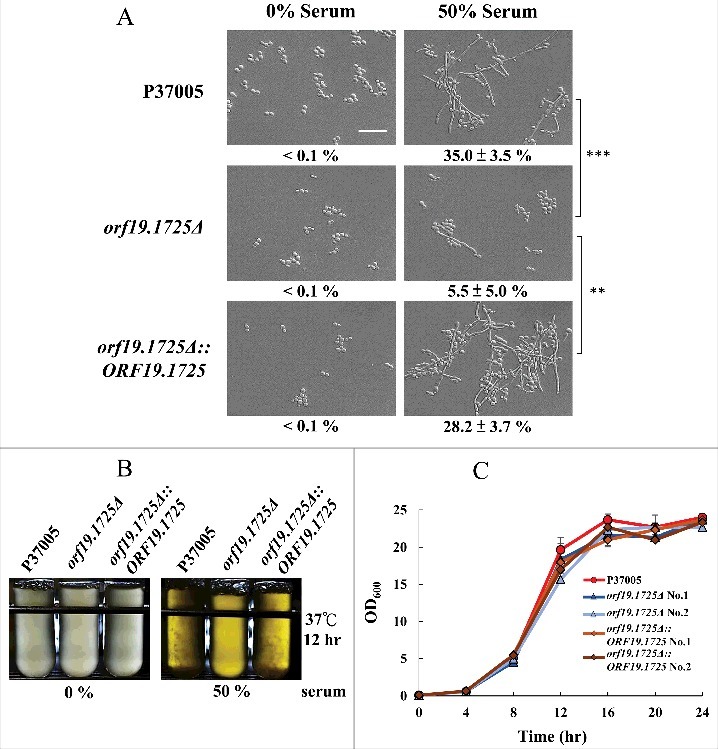
The *orf19.1725*Δ strain was defective in hyphal formation. (A) Cells of the wild-type, *orf19.1725Δ* and complemented strains were grown in YPD medium with or without 50% bovine serum. The representative images show that *ORF19.1725* gene deletion caused a severe defect in hyphal development. Four fields were checked, and at least 100 cells were counted in each field of every *C. albicans* strain. Ratios of hyphae formation are displayed below each image. “**” represents *P* < 0.01 and “***” represents *P* < 0.001. (B) The wild-type and the complemented strains clearly exhibited clumps after 12 hr of culturing in YPD liquid media at 37°C, whereas the *orf19.1725Δ* strains did not. Hyphae were observed and examined under a light microscope after the clumps had been collected by centrifugation. Left panel: without serum treatment; right panel: YPD supplemented with 50% bovine serum. Scale bar: 50 μm. (C) Growth curves of *C. albicans* strains at 37°C. Overnight cultures of YPD-grown *C. albicans* cells were diluted to an OD_600_ of 0.1 in fresh YPD liquid media. Growth rates were monitored every 4 hr using a Biowave density meter. Values are the mean ± SD of three experimental replicates, and two technical repeats were performed for each experimental replicate. “**” represents *P* < 0.01 and “***” represents *P* < 0.001.

We have shown that the presence of *ORF19.1725* is important for biofilm formation in *C. albicans* P37005. Moreover, our data showed that genetic control of *ORF19.1725* is mediated by two regulators, Cph1 and Tec1. *TEC1* deletion strains in *C. albicans* produce no biofilms and are defective in hyphal development [41]. We then examined whether *ORF19.1725* also plays a role in hyphal formation. As expected, removing *ORF19.1725* caused a significant hyphal development deficiency when cultured in 50% serum (Fig. 4A). Additionally, after 12 hr of culture in serum, both the wild-type and complemented strains displayed obvious cell accumulations and clumps (hyphal formation; Fig. 4B), whereas *orf19.1725* mutants did not. These results indicate that *ORF19.1725* is involved in hyphal development. In order to determine whether or not growth defects happen in *ORF19.1725* deletion strains, thereby causing a severe deficiency in hyphal development, growth curves of the wild-type, mutant and complementation strains were determined. Fig. 4C shows that *orf19.1725* mutant strains did not exhibit growth defects.

### *ORF19.1725* potentially encodes an adhesin protein

**Figure 5.**
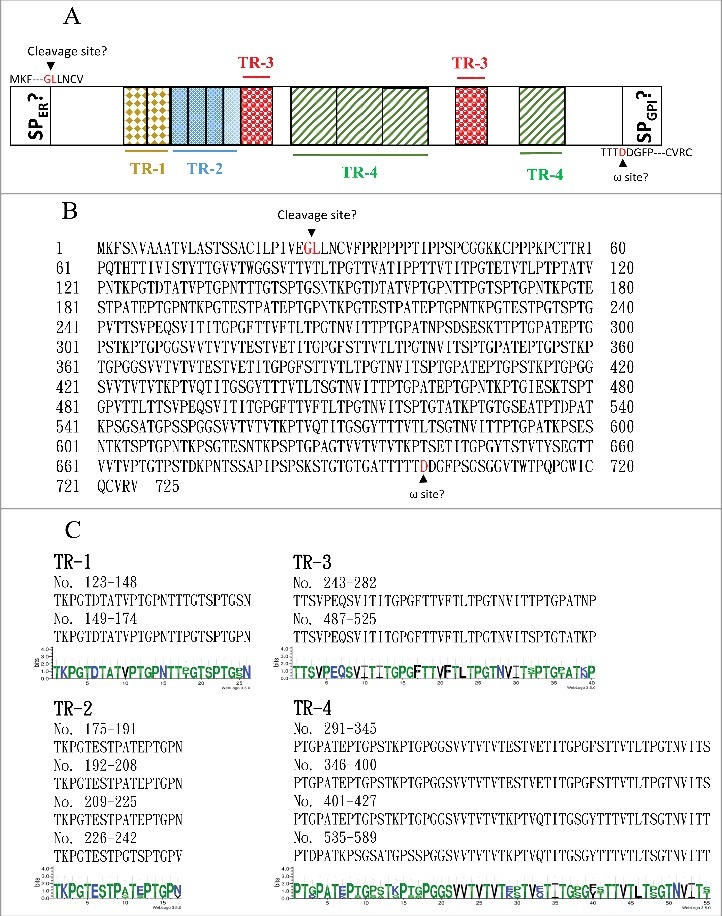
The *C. albicans* Orf19.1725 protein potentially functions as an adhesin. (A) Analysis of Orf19.1725 reveals three conserved features, a putative signal peptide, four different tandem repeats (TR1, TR2, TR3 and TR4) and a hypothetical ω site. (B) Protein sequence of *C. albicans* orf19.1725. The arrow between the 26th (Gly) and 27th (Leu) amino acids indicates the likely cleavage site for the signal peptide (http://www.cbs.dtu.dk/services/SignalP/). The other, at the position of the 700th amino acid (Asp), was predicted to be an ω site with lower specificity (http://gpcr.biocomp.unibo.it/predgpi/). (C) Sequence motifs of four different tandem repeats of Orf19.1725 (http://weblogo.threeplusone.com/create.cgi).

The major fungal adhesins are glycosylphosphatidylinositol (GPI)-anchored proteins [42]. The primary structure of these GPI-anchored proteins can be divided into three conserved features [42, 43]. The N terminus contains a signal peptide for localization to the endoplasmic reticulum (ER). Although the middle regions of adhesin proteins are diverse, they are often rich in serine/threonine residues and contain tandem repeats. The hydrophobic C-terminal domain contains an ω site, where the protein is cleaved and replaced with a preformed GPI anchor in the membrane of the ER. Orf19.1725 has been predicted to function as an adhesin protein (FungalRV; http://fungalrv.igib.res.in/immuno.html) [44], and the coding region of Orf19.1725 was therefore further analyzed. A signal peptide (SP) in the N-terminal region was predicted (value: 0.452) using SignalP 4.1 with the default cutoff value (0.450) (http://www.cbs.dtu.dk/services/SignalP/) [45], and a cleavage site was predicted between the 26th and 27th amino acids (Fig. 5A and 5B). Although an ω site at the position of the 700th amino acid was predicted by the PredGPI website (http://gpcr.biocomp.unibo.it/predgpi/), this prediction is less likely to be accurate due to a very low specificity (32.5%) (Fig. 5A and 5B) [46]. Interestingly, four tandem repeats were found (Fig. 5A and 5C): TR1 (26 aa), TR2 (17 aa), TR3 (40 aa) and TR4 (55 aa) (http://weblogo.threeplusone.com/create.cgi) [47]. These data reveal that Orf19.1725 might be an adhesin and is involved in cell adhesion. However, the protein sequence of Orf19.1725 does not share any sequence or feature similarities with the three known adhesin families (*ALS, HWP* and *IFF*) in *C. albicans* [42,48–50].

### Orf19.1725 is required for cell adhesion in a zebrafish egg infection model

**Figure 6.**
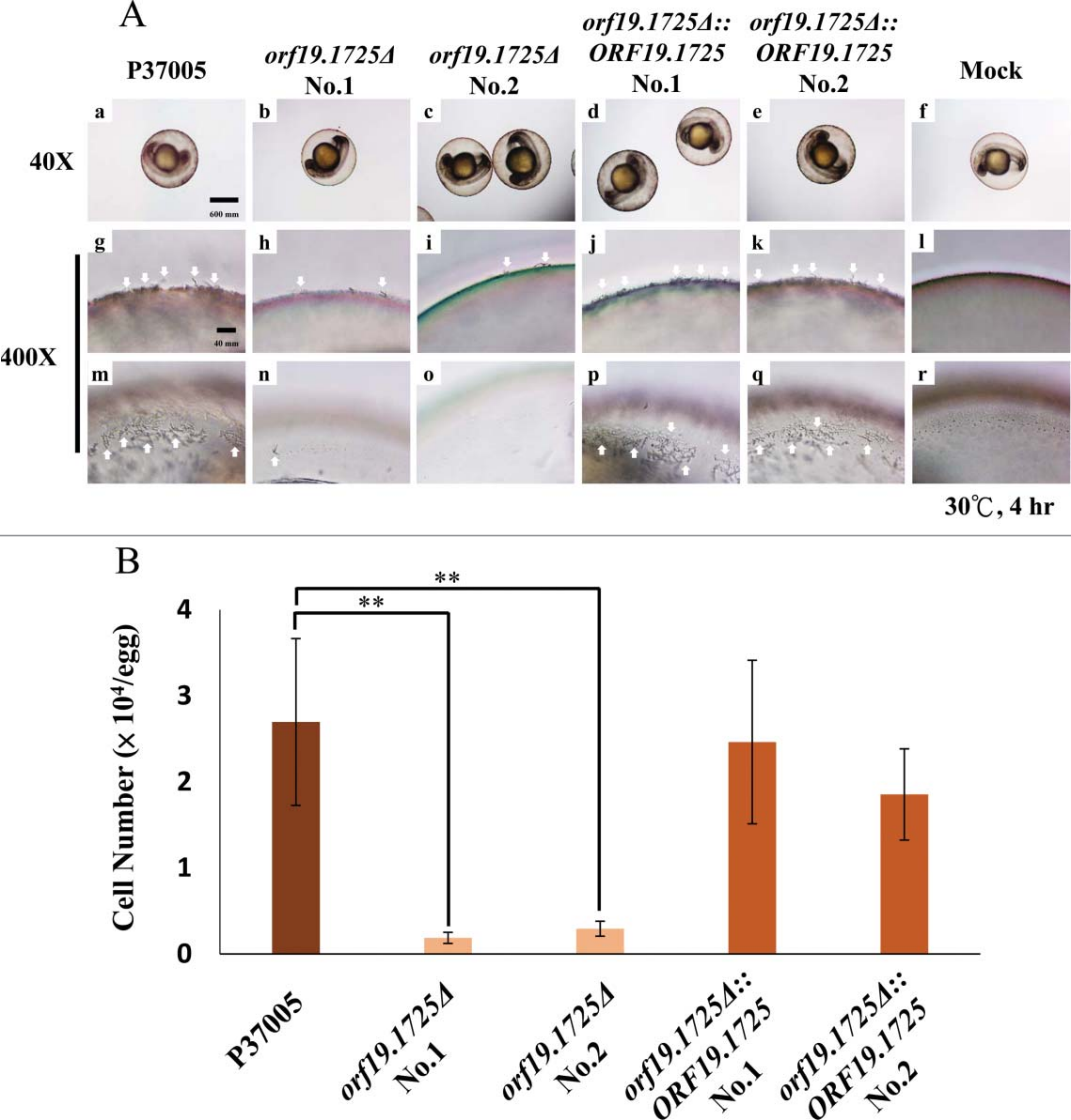
*ORF19.1725* is involved in cell adhesion to zebrafish embryos. (A) Embryos were co-incubated with 5 × 10^5^ *C. albicans* cells of P37005 (a, g, m), *orf19.1725Δ* (b, c, h, i, n, o) and *orf19.1725Δ::ORF19.1725* (d, e, j, k, p, q) for 4 hr. The infected embryos were then washed with egg water to remove non-adhered cells. Images were taken by a Leica TCS SP5 II inverted microscope. The images in the middle panel (g, h, i, j, k, l) are focused on the outside of the embryo surface. The images in the bottom panel (m, n, o, p, q, r) are focused on the inner side of the embryo surface. Embryos without infection by *C. albicans* cells served as negative controls (f, l, r). White arrows indicate hyphae on or in the embryo surfaces. (B) Cell quantitation revealed a significantly lower number of adhesive cells of *orf19.1725Δ* on zebrafish embryo surfaces than of the wild-type or the complemented strains. Infected embryos were washed twice with 40 ml of egg water to remove non-adhered cells. The embryo eggs were then disrupted by a FastPrep-24 instrument (MP Biomedicals, Illkirch, France) and plated on YPD plates to quantitate the number of adhered cells. Values are the mean ± SD of three experimental replicates, and two technical repeats were performed for each experimental replicate “**” represents *P* < 0.01.

To test whether *ORF19.1725* has a role in adhesion, cell adhesion assays using a zebrafish egg infection model were performed (Chen et al., 2015). As shown in Fig. 6A, two independent *orf19.1725* mutant strains almost entirely failed to attach to zebrafish eggs after 4 hr of inoculation (Fig. 6Ab, c, h, I), whereas the P37005 wild-type (Fig. 6Aa, g) and the complemented strains (Fig. 6Ad, e, j, k) colonized the entire surfaces of the embryo eggs successfully and formed hyphae. Furthermore, the P37005 wild type (Fig. 6Am) and the complemented strains (Fig. 6Ap and q), but not the mutant strains (Fig. 6An and o), were able to penetrate and invade the embryo eggs within the early infection stage (4-hr inoculation). This phenomenon could be caused by the presence of fewer adherent cells in *orf19.1725Δ* or by defects in hyphal formation in these mutant strains. Infected embryo eggs were then disrupted in a vortex shaker using glass beads to quantitate the adhered cells. The number of adhered cells of the *orf19.1725* mutant strains was significantly lower than those of the wild-type and complemented strains (Fig. 6B).

### *ORF19.1725* gene deletion significantly reduced fungal virulence in P37005

**Figure 7.**
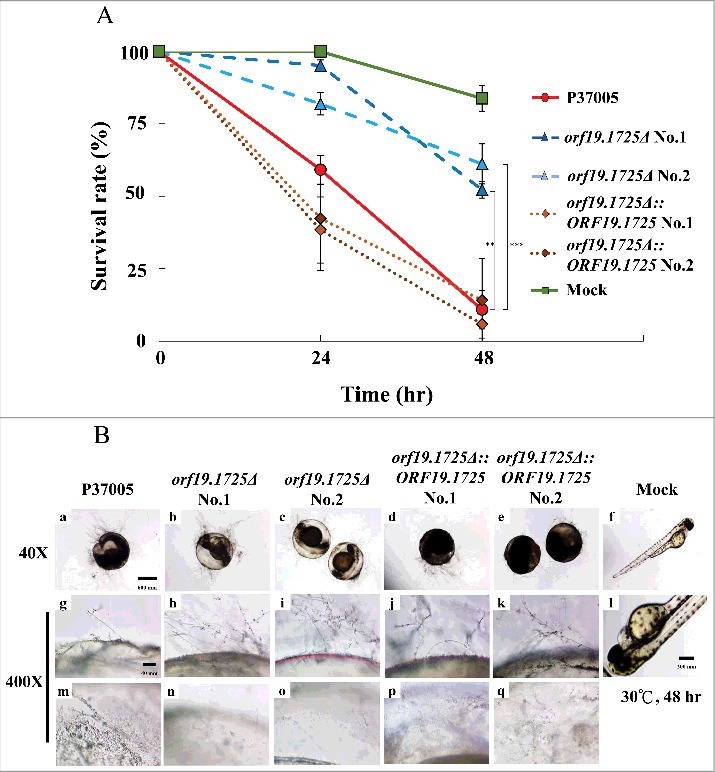
*ORF19.1725* deletion strains had reduced virulence. (A) Survival curves of zebrafish embryos during infection with *C. albicans* cells revealed that *Orf19.1725* is involved in pathogenicity. Twenty embryos in each plastic well were co-incubated with 5 × 10^5^ *C. albicans* cells. Infected embryos were washed and transferred to sterilized egg water. Survival rate was determined after calculating the number of infected zebrafish embryos that retained a heartbeat divided by the total embryo number. Values are the mean ± SD of three experimental replicates, and each replicate represents two technical repeats. “**” represents *P* < 0.01 and “***” represents *P* < 0.001. (B) Representative images of embryos infected by *C. albicans* after 48 hr revealed that *orf19.1725Δ* exhibited slower infection progress, less penetration ability and a clearer embryo background (b, c, h, i, n, o) than the wild-type strain (a, g, m) and the complemented strains (d, e, j, k, p, q) did. Embryos without infection by *C. albicans* cells served as mock controls (f, l) and hatched into newborn zebrafish. The images in the middle panel (g, h, i, j, k, l) are focused on the outside of the embryo surface. The images in the bottom panel (m, n, o, p, q, r) are focused on the inner side of the embryo surface.

We have shown that the *orf19.1725Δ* strain had a defect in hyphal formation under serum treatment and decreased adhesion ability on zebrafish embryos. To further evaluate the role of *ORF19.1725* in virulence, the survival rates of zebrafish embryos at 24 and 48 hr post-inoculation were determined. As shown in Fig. 7A, *ORF19.1725* deletion strains resulted in higher embryo survival rates at 24 hr (95.1% and 81.9%) and at 48 hr (53.1% and 61.1%) after infection. In contrast, the P37005 wild-type (61.0%) and the complemented strains (41.5% and 38.4%) had lower embryo survival than that of *orf19.1725Δ* at 24 hr. Few embryos survived after inoculation with the wild-type (10.9%) and complemented strains (14.9% and 5.8%) at 48 hr. Although microscopy showed that *orf19.1725Δ* had normal hyphal development on infected embryos at 48 hr (Fig. 7Bb, c, h, i), similar to that of the wild-type (Fig. 7Ba, g) and complemented strains (Fig. 7B d, e, j, k), the *ORF19.1725* deletion strains showed remarkable defects in penetration ability (Fig. 7Bb, c, n, o). The inner sides of the embryo surfaces showed that few hyphae had penetrated (Fig. 7Bn, o), leading to a clearer background (Fig. 7Bb, c) in the embryos infected with *orf19.1725Δ*, whereas the hyphae produced by the wild-type (Fig. 7Ba, m) and complemented strains (Fig. 7Bd, e, p, q) had successfully invaded the embryos.

## Discussion

The epigenetic transition between white cells and opaque cells is a unique phenomenon in *C. albicans* [8,19,20]. White cells and opaque cells not only display distinct cellular appearances and sizes but also exhibit a wide variety of changes in different aspects of their biology, including virulence, biofilm formation, fungus-host interactions and mating [5,10,15,17,31–33,38]. In particular, the pheromone responses in white and opaque cells are behaviorally distinct, in that opaque cells mate, whereas white cells do not mate but instead initiate cell adhesion [31]. Strikingly, expression profiling of *C. albicans* strain P37005 revealed that the components required for pheromone signaling have significant overlap with the genes regulated by Cph1 in both the white and opaque states [31]. Hence, the strategies of these two distinct cell types of *C. albicans* to determine a specific regulatory program to respond to pheromone challenge represent an interesting and crucial question.

Three potential assumptions might account for the distinct biological outputs of pheromone-stimulated white cells and opaque cells. **(1)** Although the overlap in gene expression between pheromone-stimulated white cells and opaque cells was significant, expression levels were higher overall in opaque cells than in white cells. These data imply that gene expression level might be a crucial factor in phenotype-specific response [31]. **(2)** Furthermore, differential expression levels might play a role in signaling specificity. Indeed, a recent article has shown that co-expression of *STE4, CST5*, and *CEK2* in white cells significantly increased their abilities to undergo cell-cell mating [31]. **(3)** In *S. cerevisiae*, differential Ste12-bound complexes are well known to modulate different developmental fates through the MAP kinase pathway [51,52]. During filamentation, the formation of a Ste12-Dig1-Tec1 complex is required, whereas inhibition of the Tec1 transcription factor is necessary to ensure that the sexual program occurs [51,52]. Thus, different Cph1-biding partners likely contribute to target gene binding specificity, leading to different biological responses in *C. albicans* white cells and opaque cells.

In this study, five genes with significantly higher expression levels in white cells than in opaque cells were selected for further investigation. Four of these five genes, *ORF19.1539, ORF19.1725, ORF19.2430* and *ORF19.5557*, are specifically involved in the pheromone response in white cells, as deletion of each gene significantly reduced pheromone-stimulated cell adhesion but did not affect normal mating ability. These results demonstrate for the first time that a particular set of genes regulated by Cph1 is specifically involved in the white cell pheromone response and differentiates that response from the opaque cell pheromone response.

Genetic control of pheromone-stimulated cell adhesion and conventional biofilm formation in *C. albicans* is mediated by different mechanisms. In particular, Cph1 is required for pheromone-induced cell adhesion in white cells [31], whereas six transcriptional regulators (Bcr1, Brg1, Efg1, Rob1, Ndt80 and Tec1) have been shown to operate in the biofilm regulatory network [34]. Although Bcr1, Brg1, Rob1 and Tec1 play general roles in mediating pheromone-stimulated cell adhesion as well as in biofilm development [31,32,34], *CPH1* deletion strains in *C. albicans* produce conventional biofilms normally but are completely deficient in pheromone-stimulated cell adhesion [31]. We have previously shown that Hgc1, a G1 cyclin-related protein regulated by both Cph1 and Tec1, is important for both pheromone-stimulated cell adhesion and biofilm development [31]. Here, we have shown that *ORF19.1725* is also involved in white cell pheromone response and plays a critical role in conventional biofilm formation in *C. albicans* P37005. Indeed, *ORF19.1725* is also regulated by Tec1. Cells lacking *ORF19.1725* exhibit a marked defect in hyphal formation, which may explain the inability of *orf19.1725Δ* to form conventional biofilms.

Sequence analyses showed that Orf19.1725 might function as an adhesin protein. The results of zebrafish egg adhesion assays demonstrated that *orf19.1725Δ* had reduced adhesive ability on the surfaces of zebrafish embryos. However, expression of *ORF19.1725* fused with *green fluorescent protein* (*GFP)* at either the N- or C-terminal end failed to determine its subcellular localization (data not shown). As described previously, an adhesin protein has an SP for entry into the ER and a cleavage ω site for anchoring to a preformed GPI [42,43]. Thus, the *GFP* fused with the N- and C-terminal sites of Orf19.1725 was likely removed during processing. Insertion of this fluorescent marker into the signal cleavage site or the ω site might be a better strategy to determine its localization. However, whether the SP cleavage site at the N terminus and the ω site at the C terminus of Orf19.1725 are accurate in *C. albicans* needs to be further addressed, given that the prediction values for both sites were low.

Our results have shown that *ORF19.1725* is required for hyphal development and biofilm formation *in vitro* as well as cell adhesion *in vivo*. As expected, *ORF19.1725* deletion also resulted in a significant decrease in virulence. However, hyphal development and hyphal length between the wild-type and *orf19.1725Δ* strains were indistinguishable in the zebrafish egg infection model at 48 hr post-inoculation. The distinct hyphal development phenotypes of *orf19.1725Δ* observed *in vitro* and *in vivo* might be due to different environmental conditions. In fact, a variety of hyphal stimulators, such as high temperature, serum, neutral pH and nutrient limitation can promote the yeast-to-hyphae transition through different signaling pathways and transcription factors [7, 53–56].

Despite utilizing the same signaling cascade and transcription factors, white cells and opaque cells exhibit distinct biological outputs in response to pheromone stimulation. Here, we have shown that some pheromone-induced white-specific genes contribute to the phase-specific phenotype of pheromone-responding white cells in *C. albicans* P37005. Notably, deletion of *ORF19.1725* resulted in decreased pheromone-induced cell adhesion in pheromone-responding white cells and abolished biofilm formation, similar to *tec1Δ* and *hgc1Δ* [31,41]. Additionally, we have also characterized and determined new roles of *C. albicans ORF19.1725* in cell adhesion, hyphal formation and virulence. Most importantly, the results outlined in this study might provide a clue as to how the molecular switch regulates the alternative phenotypic outputs of white cells and opaque cells during pheromone response, with direct consequences for pathogenesis and sex.

## Materials and methods

### Media and reagents

The media used in these experiments, including yeast extract-peptone-dextrose (YPD), Spider medium and Lee's medium, were prepared as previously described [57]. YPD medium, composed of 2% (w/v) peptone, 1% (w/v) yeast extract, and 2% (w/v) glucose, was used routinely for strain growth and maintenance. YPD medium containing 200 μg/ml of nourseothricin (Werner BioAgents, Jena, Germany) was used to select nourseothricin-resistant strains. Spider medium (pH 7.2), containing 1% (w/v) mannitol, 1% (w/v) nutrient broth, and 0.4% (w/v) dipotassium phosphate (Showa Chemical Industry, Japan), was used for opaque cell maintenance. All chemicals were purchased from Sigma-Aldrich Chemical, unless otherwise stated.

Growth curves were performed as described previously with slight modifications [58]. We measured the growth curves using a Biowave density meter (WPA CO8000). *C. albicans* strains were grown overnight in YPD, diluted to an optical density at 600 nm (OD_600_) of 0.1 in fresh YPD and incubated at 30°C and 100 rpm. The optical density was evaluated every 4 hr.

### Plasmid and strain construction

*C. albicans* strains and oligonucleotides used in this study are listed in Tables S1 and S2, respectively. To generate each mutant strain, the 5**′** flanking and 3**′** flanking regions of each gene were PCR amplified using different primer sets and individually cloned into pSFS2A [59]. These constructs were linearized with restriction enzymes and transformed into *C. albicans* to generate each deletion mutant strain.

To generate the *orf19.1539Δ* strains, the 5**′** flanking and 3**′** flanking regions of *ORF19.1539* were PCR amplified using primers 395/396 and 397/398, respectively. The 5**′** and 3**′** PCR products were digested with *Apa*I/*Xho*I and *Sac*II/*Sac*I, respectively, and cloned into the plasmid pSFS2A to generate the plasmid pSFS2A-orf19.1539 KO. The plasmid was digested with *Apa*I/*Sac*I and transformed into the wild-type strain P37005 to generate heterozygous *orf19.1539Δ*/*ORF19.1539* mutants. The *SAT1* marker was recycled by treatment with 2% maltose. The heterozygous strains were retransformed with the same deletion construct to generate the *orf19.1539Δ/orf19.1539Δ* strains YL1031/YL1032.

To generate *orf19.1725Δ* strains, the 5**′** flanking and 3**′** flanking regions of *ORF19.1725* were PCR amplified using primers 363/364 and 365/366, respectively. The 5**′** and 3**′** PCR products were digested with *Apa*I/*Xho*I and *Sac*II/*Sac*I, respectively, and cloned into the plasmid pSFS2A to generate the plasmid pSFS2A-orf19.1539 KO. The plasmid was digested with *Apa*I/*Sac*I and transformed into P37005 to generate heterozygous *orf19.1725Δ*/*ORF19.1725* mutants. The *SAT1* marker was recycled, and the strains were retransformed with the same construct to generate *orf19.1725Δ*/*orf19.1725Δ* strains YL1033/YL1034. The *ORF19.1725* complementation construct was made by amplification of its endogenous promoter and open reading frame (ORF) using primer 647/669. The PCR product was digested with *Apa*I/*Xho*I and cloned into pSFS-orf19.1725 KO to generate pSFS-orf19.1725AB. The plasmid was digested with *Apa*I/*Sac*I and transformed into *orf19.1725Δ* to create YL1241/YL1242.

To generate *orf19.2430Δ* strains, the 5**′** flanking and 3**′** flanking regions of *ORF19.2430* were PCR amplified using primers 323/324 and 325/326, respectively. The 5**′** and 3**′** PCR products were digested with *Apa*I/*Xho*I and *Sac*II/*Sac*I, respectively, and cloned into the plasmid pSFS2A to generate the plasmid pSFS2A-orf19.2430 KO. The plasmid was digested with *Apa*I/*Sac*I and transformed into P37005 to generate heterozygous *orf19.2430Δ*/*ORF19.2430* mutants. Homozygous *orf19.2430Δ*/*orf19.2430Δ* strains YL1037/YL1038 were created after the *SAT1* marker was removed and the strains were retransformed with the same deletion construct.

To generate *orf19.2691Δ* strains, the 5**′** flanking and 3**′** flanking regions of *ORF19.2691* were PCR amplified using primers 451/452 and 453/454, respectively. The 5**′** and 3**′** PCR products were digested with *Apa*I/*Xho*I and *Not*I/*Sac*II, respectively, and cloned into the plasmid pSFS2A to generate the plasmid pSFS2A-orf19.2691 KO. The plasmid was digested with *Apa*I/*Sac*I and transformed into *MTL*a/a to generate heterozygous *orf19.2691Δ*/*ORF19.2691* mutants. The *SAT1* marker was recycled and the strains retransformed with the same deletion construct to generate *orf19.2691Δ/orf19.2691Δ* strains YL1067/YL1068.

To generate *orf19.5557Δ* strains, the 5**′** flanking and 3**′** flanking regions of *ORF19.5557* were PCR amplified using primers 331/332 and 333/334, respectively. The 5**′** and 3**′** PCR products were digested with *Apa*I/*Xho*I and *Sac*II/*Sac*I, respectively, and cloned into the plasmid pSFS2A to generate the plasmid pSFS2A-orf19.5557 KO. The plasmid was digested with *Apa*I/*Sac*I and transformed into *MTL*a/a to generate heterozygous *orf19.5557Δ*/*ORF19.5557* mutants. The *SAT1* marker was recycled and the strains retransformed with the deletion construct to generate *orf19.5557Δ/orf19.5557Δ* strains YL1087/YL1088.

Opaque cells of each strain were obtained and purified after treatment with Lee's NAG (Table S1).

### Hyphal formation tests

Overnight culture of the wild-type P37005, the *orf19.1725* mutant strains and the complemented strains were grown in YPD supplemented with 0% serum or 50% serum for 12 hr. The hyphal development of each sample in different culture conditions was examined by the use of an Eclipse Ti inverted microscope (Nikon Instruments Inc., Melville, NY, USA).

### Pheromone-stimulated cell adhesion assays

Approximately 5 × 10^7^ cells of overnight cultures from Spider medium were added to 1 ml of Lee's medium with or without 0.01% dimethyl sulfoxide (DMSO) or 10 μM α-pheromone (GFRLTNFGYFEPG; synthesized by GMbiolab) in a 12-well plastic plate (Costar, Corning) [60]. The inoculated plates were incubated at room temperature for 16 hr without shaking. Each sample was washed with phosphate-buffered saline (PBS) to remove the non-adhered cells. The samples were then photographed, and the adhered cells were scratched out of the plastic well using a sterilized tip and quantified by measuring the OD_600_. The experiment was performed in three experimental replicates with each replicate containing two technical repeats

### Conventional biofilm assay

Conventional biofilm assays were performed based on the previously established protocol to measure fungal mass in a silicone model [34]. Sterile silicone squares (Bentec Medical, PR72034-06N, 1.5 cm × 1.5 cm) were weighed before incubating in bovine serum (Sigma B-9433) in a 12-well plastic plate. The plastic plates were shaken at 150 rpm at 37°C overnight. Each serum-treated silicone square was washed with 2 ml PBS and placed in new sterilized 12-well culture dishes containing 2 ml Spider medium. Approximately 2 × 10^7^ cells from YPD overnight cultures of the *C. albicans* P37005 wild-type and mutant strains were gently added on top of the silicone squares. Inoculated samples were incubated at 37°C and 150 rpm for 90 min for adhesion. Silicone squares were washed with 2 ml PBS and then incubated in 2 ml fresh Spider medium for 60 hr at 37°C and 150 rpm. The dry weight of the biofilm mass of each silicone square was determined after the supernatant was removed, and the silicone squares were dried overnight. The biofilm assays were performed in three experimental replicates with each replicate containing two technical repeats.

### Quantitative mating assays

The opaque P37005 *MTL***a/a** wild-type strain and mutant strains (SAT^−^, Arg^+^) and the *MTL*α/α DSY211 strain (SAT^+^, Arg^−^) [40] were grown in Spider medium at room temperature [57]. Approximately 2 × 10^7^ *MTL***a/a** and *MTL*α/α cells were mixed together on a nitrocellulose filter on Spider medium. Cells were incubated for 48 hr at 25°C and then resuspended in 1 ml sterilized ddH_2_O and plated onto selective media (SAT^+^, Arg^−^) to quantitate mating frequency, as previously described [34]. The experiment was done in three experimental replicates with each replicate containing two technical repeats.

### Adhesion assays using zebrafish egg

Zebrafish embryos were generated from zebrafish mating as described previously [61]. Briefly, zebrafish embryos were collected after male and female zebrafishes mated. Embryos were then sterilized using 0.028% chlorine bleach containing 0.0017% sodium hypochlorite. The bleach solution was removed, and the embryos were washed twice in 40 ml sterilized ddH_2_O. The embryos were bleached again and washed with egg water (0.03% sea salt) 3 times. Twenty embryos were transferred into each well of a 24-well plate with Roswell Park Memorial Institute 1640 medium (RPMI). A total of 5 × 10^5^ *C. albicans* cells of the wild-type P37005 strain and the *orf19.1725Δ* strains were co-incubated with embryos for 4 hr at 30°C. The embryos were then transferred into conical tubes containing 40 ml egg water and washed twice to remove non-adhered cells at 100 rpm for 3 min. The embryos were transferred into a new 24-well plate containing 0.7 ml egg water with 0.5% YPD. A Leica TCS SP5 II inverted microscope was used for confocal imaging. To quantitate adherent cells, embryos were sacrificed, disrupted by a FastPrep-24 instrument (MP Biomedicals, Illkirch, France), diluted and plated on YPD plates, and the number of CFUs was counted. The experiment was performed in three experimental replicates with each replicate containing two technical repeats. Statistical significance was determined using Student's t-test.

### Virulence of *C. albicans* using zebrafish egg infection model

Twenty embryos were co-incubated with 5 × 10^5^ *C. albicans* cells in 6-well plates containing 4 ml of RPMI serum at 30°C with shaking at 80 rpm for 4 hr for each experiment [61]. Non-adhered *C. albicans* cells were removed, and the embryos were incubated in egg water. To determine whether embryos were dead or alive, a Leica TCS SP5 II inverted microscope was used to confirm whether the infected embryo's heart was still beating. Survival rate was determined by calculating the number of infected zebrafish embryos that still had heartbeats divided by the total embryo number in each well after 24 hr and 48 hr of incubation at 30°C. Virulence assays were performed in three experimental replicates with each replicate containing two technical repeats. Statistical significance was determined using Student's t-test.

### Statistical analyses

All statistical analyses were performed with Excel software. Differences between the wild-type and mutant strains were analyzed using a one-tailed, two-typed Student's *t*-test with a 95% confidence interval. All the experiments were performed in three experimental replicates with each replicate containing two technical repeats. The data are presented as the mean ± SD, and *P* < 0.05 was considered statistically significant.

## Supplementary Material

Table_S1_and_S2_12-1.docx
