# Supplementary material for: Identification and characterization of ORF19.1725, a novel gene contributing to the white cell pheromone response and virulence-associated functions in Candida albicans
Source: Virulence. 2018 May 4;9(1):866–78. doi: 10.1080/21505594.2018.1456228 (PMC5955465; doi:10.1080/21505594.2018.1456228)
Supplement: Table_S1_and_S2_12-1.docx [file kvir-09-01-1456228-s001.docx]

**Table S1. Strains used in this study.**

| **Strain** | ***MTL* type** | **Genotype** | **White or opaque** | **Source** |
| --- | --- | --- | --- | --- |
| P37005 | **a**/**a** | Wild-type | White | [[1](#_ENREF_1)] |
| DSY211 | α/α | *leu2::hisG/leu2::hisG his1::hisG/his1::hisG::SAT1* | Opaque | [[2](#_ENREF_2)] |
| YL224 | **a**/**a** | *cph1*/*cph1* | White | [[3](#_ENREF_3)] |
| YL225 | **a**/**a** | *cph1*/*cph1* | White | [[3](#_ENREF_3)] |
| YL242 | **a**/**a** | *tec1*/*tec1* | White | [[3](#_ENREF_3)] |
| YL243 | **a**/**a** | *tec1*/*tec1* | White | [[3](#_ENREF_3)] |
| YL1031 | **a**/**a** | *orf19.1539*/*orf19.1539* | White | This study |
| YL1032 | **a**/**a** | *orf19.1539*/*orf19.1539* | White | This study |
| YL1033 | **a**/**a** | *orf19.1725*/*orf19.1725* | White | This study |
| YL1034 | **a**/**a** | *orf19.1725*/*orf19.1725* | White | This study |
| YL1037 | **a**/**a** | *orf19.2430*/*orf19.2430* | White | This study |
| YL1038 | **a**/**a** | *orf19.2430*/*orf19.2430* | White | This study |
| YL1067 | **a**/**a** | *orf19.2691*/*orf19.2691* | White | This study |
| YL1068 | **a**/**a** | *orf19.2691*/*orf19.2691* | White | This study |
| YL1087 | **a**/**a** | *orf19.5557*/*orf19.5557* | White | This study |
| YL1088 | **a**/**a** | *orf19.5557*/*orf19.5557* | White | This study |
| YL1241 | **a**/**a** | *orf19.1725*/*orf19.1725::ORF19.1725* | White | This study |
| YL1242 | **a**/**a** | *orf19.1725*/*orf19.1725::ORF19.1725* | White | This study |
| YL1619 | **a**/**a** | *orf19.1725*/*orf19.1725* | Opaque | This study |
| YL1629 | **a**/**a** | *orf19.1725*/*orf19.1725* | Opaque | This study |
| YL1719 | **a**/**a** | *orf19.1539*/*orf19.1539* | Opaque | This study |
| YL1720 | **a**/**a** | *orf19.1539*/*orf19.1539* | Opaque | This study |
| YL1721 | **a**/**a** | *orf19.2430*/*orf19.2430* | Opaque | This study |
| YL1722 | **a**/**a** | *orf19.2430*/*orf19.2430* | Opaque | This study |
| YL1727 | **a**/**a** | *orf19.5557*/*orf19.5557* | Opaque | This study |
| YL1728 | **a**/**a** | *orf19.5557*/*orf19.5557* | Opaque | This study |
| YL1775 | **a**/**a** | *cph1*/*cph1* | Opaque | This study |
| YL1776 | **a**/**a** | *cph1*/*cph1* | Opaque | This study |

References

1. Daniels KJ, Srikantha T, Lockhart SR, et al. Opaque cells signal white cells to form biofilms in *Candida albicans*. EMBO J. 2006;25(10):2240-52. doi: 10.1038/sj.emboj.7601099.

2. Alby K, Bennett RJ. Interspecies pheromone signaling promotes biofilm formation and same-sex mating in *Candida albicans*. Proceedings of the National Academy of Sciences of the United States of America. 2011;108(6):2510-5. doi: 10.1073/pnas.1017234108.

3. Lin CH, Kabrawala S, Fox EP, et al. Genetic control of conventional and pheromone-stimulated biofilm formation in *Candida albicans*. PLoS Pathog. 2013;9(4):e1003305. doi: 10.1371/journal.ppat.1003305.

**Table S2. Oligonucleotides used in this study.**

| **Name** | **Sequence (5′->3′)** |
| --- | --- |
| 323 | GGAGC GGGGC CCCTG AAGAT GGAAG TACGC TTGG |
| 324 | GGAGC GCTCG AGATT AATGG TGCGA TTGAT AGTTG |
| 325 | GGAGC GCCGC GGCAA TTCCC TCGGA TTTAC TAGC |
| 326 | GGAGC GGAGC TCCAA TTCCT CGGTC ATAGA AGGG |
| 327 | CAATC AACAT CATTC TTCAA AAATG |
| 328 | CTCAT CTTTC CTTGT TTGTC AGC |
| 329 | GAGTT ATTGC ATTTT CCACC G |
| 330 | AAATT TGGAA TTAAG GTCGC TT |
| 331 | GGAGC GGGGC CCGCA GCCAA GAAAT ACCGC TATC |
| 332 | GGAGC GCTCG AGTGG CACCT TCAAC GGTTG ATAT |
| 333 | GGAGC GCCGC GGAAG GGAGA CACGG AAATG TCTAT |
| 334 | GGAGC GGAGC TCTGC GTCTC AAGGA TCACC TAAT |
| 335 | GGAAT CTTCT GGGTA ACATG ACAT |
| 336 | TGAAG CTAGT GCAGC AGCAG C |
| 337 | GGGAT CATTA CCTGG ATTAT GC |
| 338 | CCCAT TATCA TCAAA CAAAA CTTG |
| 363 | GGAGC GGGGC CCGGT ATGCT GTGGG TGCGT AAAT |
| 364 | GGAGC GCTCG AGCAG TGATG GAGTG GGTGA AGAA |
| 365 | GGAGC GCCGC GGCGG TTTGG AAATT TAATT GTTTC |
| 366 | GGAGC GGAGC TCGCC ACCGC ATTTG GTAGC AT |
| 367 | ACAGA CCTCC ACATA TCATC TTCC |
| 368 | CTTTC CATAG ATGTT TATTA GACAC G |
| 369 | GGTCC AGTCA CCACC CTTAC C |
| 370 | ACCAC CACTA CCACT TGGGA |
| 395 | GGAGC GGGGC CCCCA AACCA ATGGA TGGAC TAAC |
| 396 | GGAGC GCTCG AGGTT TGACC TCAAC AAATG ACAGC |
| 397 | GGAGC GCCGC GGCGG AGTAT TGTCC TATAG TTGCC |
| 398 | GGAGC GGAGC TCTCA GCAAT AACTG TCAAG GGG |
| 399 | CTTTC AACAC ACCAT GCACA CC |
| 400 | CGAGA AGACG AAACA TGATG ATG |
| 401 | ACTGC TACCC CTACA ACTAC CC |
| 402 | CTTTT CTTAC GACGT ATAAT ACCCC |
| 451 | GGAGC GGGGC CCCAT ACCAC TCGGA TTACC GAC |
| 452 | GGAGC GCTCG AGTGA TGTTG ATTTG GGTGT CC |
| 453 | GGA GCG GCG GCC GCT GTC GTC ACA ACA CAA GCT CTA |
| 454 | GGAGC GCCGC GGCCC CACGC TTCTT CAAAA GT |
| 455 | CAGAA ACAGT GAAAA ATGAT GCA |
| 456 | GGAAG TAGAA CTAGA AGCCC TTTTC |
| 457 | ACTCA TGAAG TTGAT GACCA GG |
| 458 | ACCAT AAAAG AACCG ATAAT GGG |
| 647 | GGAGC GCCCG GGTCC AAACC GAAAT AATTA ACAA |
| 669 | GGAGC GGGCC CTACT ATACT GGTAA ACAGT TAATA |
| 1241 | AGTGG GGAAA AGTAA ACGGA |
| 1242 | AGGGG TAGCA GTAAG GTTGA |
| 1243 | GTCCA CCTCC AAAAC CATGT |
| 1244 | CCAGG TGACA ACACC AGTAG |
| 1245 | CCGAT TAGAA GGGAA ATCGC A |
| 1246 | TTGAA GTGGC TACAA TCGCA |
| 1247 | CGATC GATCC CACCA ACAAT |
| 1248 | ATGCT CGTGT AAGGT TTGGG |
| 1249 | TAACC CTGGT GACCC AAGAA |
| 1250 | GCACC ATTAA AGAAT CGCCA |
